# Ablation of a Para-Hisian Accessory Pathway from the Non-coronary Cusp: A Case Report

**DOI:** 10.19102/icrm.2026.17087

**Published:** 2026-08-15

**Authors:** Marina Marins, Vidal Essebag, Martin Bernier

**Affiliations:** 1 McGill University Health Centre, Montreal, Canada

**Keywords:** Ablation, non-coronary cusp, para-Hisian accessory pathway, pre-excited atrial fibrillation, supraventricular tachycardia

## Abstract

Para-Hisian accessory pathways (PHAPs) remain among the most challenging substrates for catheter ablation because of their close anatomical relationship to the atrioventricular (AV) conduction system and the consequent risk of iatrogenic AV block. The non-coronary cusp (NCC) has emerged as a valuable alternative when right-sided ablation is ineffective or associated with an unacceptable risk of injury to the His bundle. We report the case of a 23-year-old man who presented with pre-excited atrial fibrillation. Electrophysiological study localized an anteroseptal/para-Hisian accessory pathway. Electroanatomical mapping identified the earliest ventricular activation at the para-Hisian region; however, multiple irrigated radiofrequency applications from the right atrial septum failed to eliminate pre-excitation because of the close proximity to the His bundle. A retrograde aortic approach to the NCC was subsequently undertaken using three-dimensional electroanatomical mapping integrated with intracardiac echocardiography. Mapping within the NCC demonstrated an accessory pathway potential with atrioventricular fusion and ventricular activation preceding delta-wave onset by 20 ms. Radiofrequency delivery at this site resulted in elimination of pre-excitation within 3 s while preserving normal AV conduction. No recurrence of pre-excitation or impairment of AV nodal conduction was observed during post-ablation assessment. This case supports the NCC as a safe and effective alternative target when conventional right-sided ablation is unsuccessful or poses a significant risk of AV conduction injury.

## Introduction

Radiofrequency catheter ablation is well established as a definitive therapy of accessory pathways (APs). Nevertheless, ablation of para-Hisian accessory pathways (PHAPs) remains challenging due to the close anatomical proximity to the normal conduction system and the associated risk of complete atrioventricular (AV) block (AVB). Current data suggest that ablation from the non-coronary cusp (NCC) can be both effective and safe. However, larger studies evaluating the long-term safety and efficacy of catheter ablation at the NCC are still lacking. This case report describes a successful anteroseptal AP ablation performed from the NCC.

## Case presentation

A 23-year-old man, with no significant past medical history, woke up at 7 a.m. with the sudden onset of palpitations and dizziness. Upon presentation to the emergency department, the electrocardiogram (ECG) revealed an irregular wide QRS complex tachycardia (left bundle branch block pattern) alternating with narrow QRS complex tachycardia at 160 bpm, with a shortest R–R interval of 200 ms. His blood pressure was stable, and an intravenous bolus of 150 mg of amiodarone was administered, followed by an intravenous maintenance dose with minimal effect. A procainamide drip was then initiated and resulted in a decrease in heart rate, narrowing the QRS complex, which was compatible with atrial fibrillation at 130 bpm. The arrhythmia terminated a few minutes after the administration of procainamide. The ECG in sinus rhythm demonstrated a short P–R interval of 100 ms and pre-excited QRS. Due to the first presentation with pre-excited atrial fibrillation and the risk of a rapid ventricular response, the patient was subsequently referred for AP ablation.

**Figure 1:**
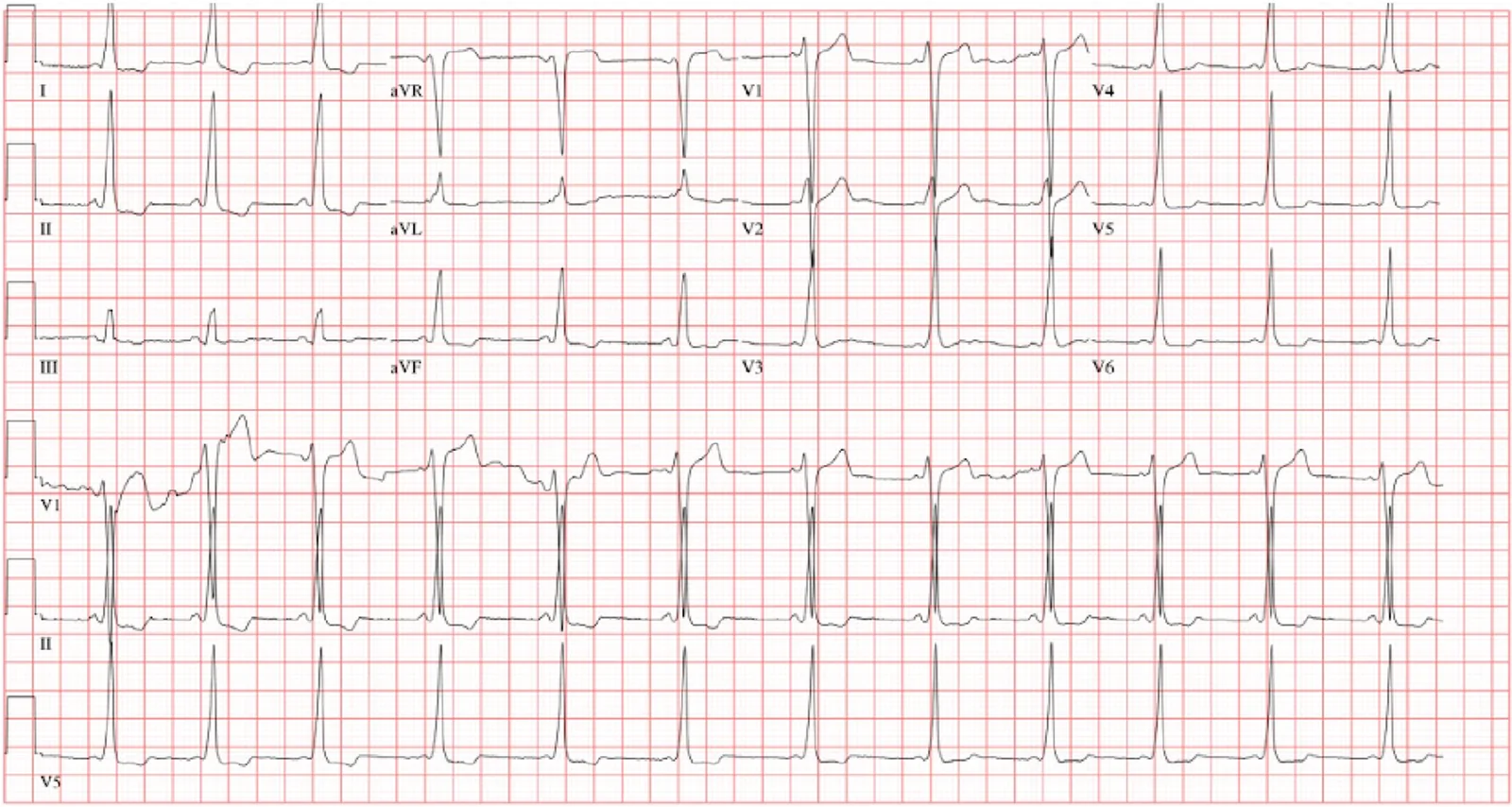
Electrocardiogram after pharmacological cardioversion demonstrating delta waves suggestive of a right anteroseptal accessory pathway.

**Figure 2:**
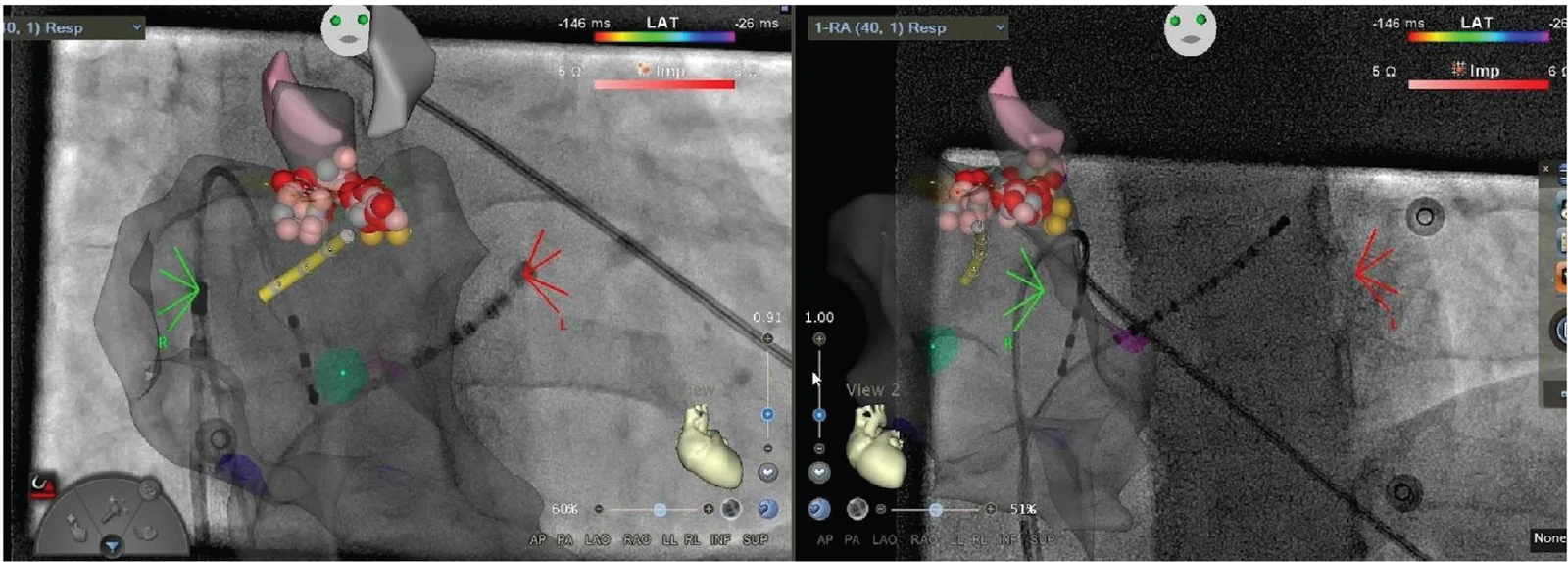
Three-dimensional mapping using CARTO™ and UniVu™; shown here is the site of initial ablation on the right anterior septal region, showing its proximity to the His region. The red dots represent the radiofrequency applications, and the yellow dots represent where His signals were identified. On the left is the right anterior oblique view and on the right is the left anterior oblique view.

**Figure 3:**
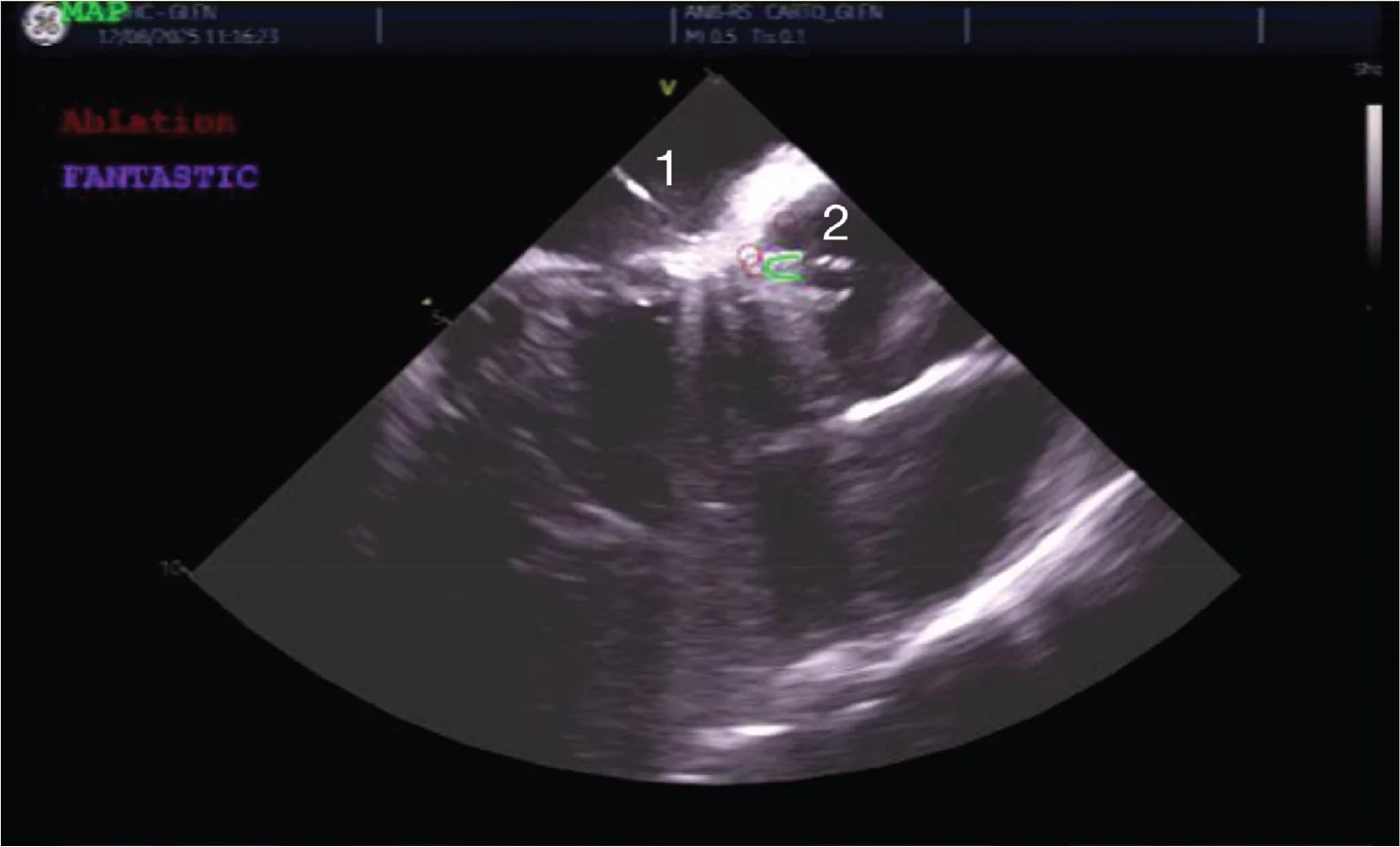
Intracardiac echocardiography showing the quadripolar catheter (1) located in the right atrium at the His region just across the non-coronary cusp (NCC) and the ablation catheter (2) positioned at the NCC of the aortic valve.

At the beginning of the procedure, the patient presented in sinus rhythm, with the baseline ECG showing a small R and slightly positive delta wave and a negative QRS in lead V1, early transition in lead V2, and a positive delta wave in the inferior leads, compatible with an anteroseptal AP **(Figure 1)**. Right femoral venous access was obtained, and a decapolar catheter was positioned in the coronary sinus (CS) while a quadripolar catheter was positioned in the His bundle and subsequently in the right ventricle for the electrophysiology study (EPS). For the mapping system, we used the CARTO™ 3 electroanatomic mapping system with CARTOSOUND™ and UniVu™ modules (J&J MedTech, New Brunswick, NJ, USA) **(Figures 2, 3, and 6)**. The baseline H–V interval was 6 ms, and the EPS demonstrated concentric ventriculoatrial (VA) conduction and confirmed the presence of an AP with an anterograde effective refractory period of 310 ms. Mapping in sinus rhythm along the tricuspid annulus identified the earliest ventricular activation and a fused atrial–ventricular potential in a para-Hisian location, anterolateral to the His. A few radiofrequency applications at this site using an irrigated-tip ablation catheter at 30 W for 30–40 s, with a temperature limit of 55°C, produced no effect on pre-excitation **(Figure 2)**.

**Figure 4:**
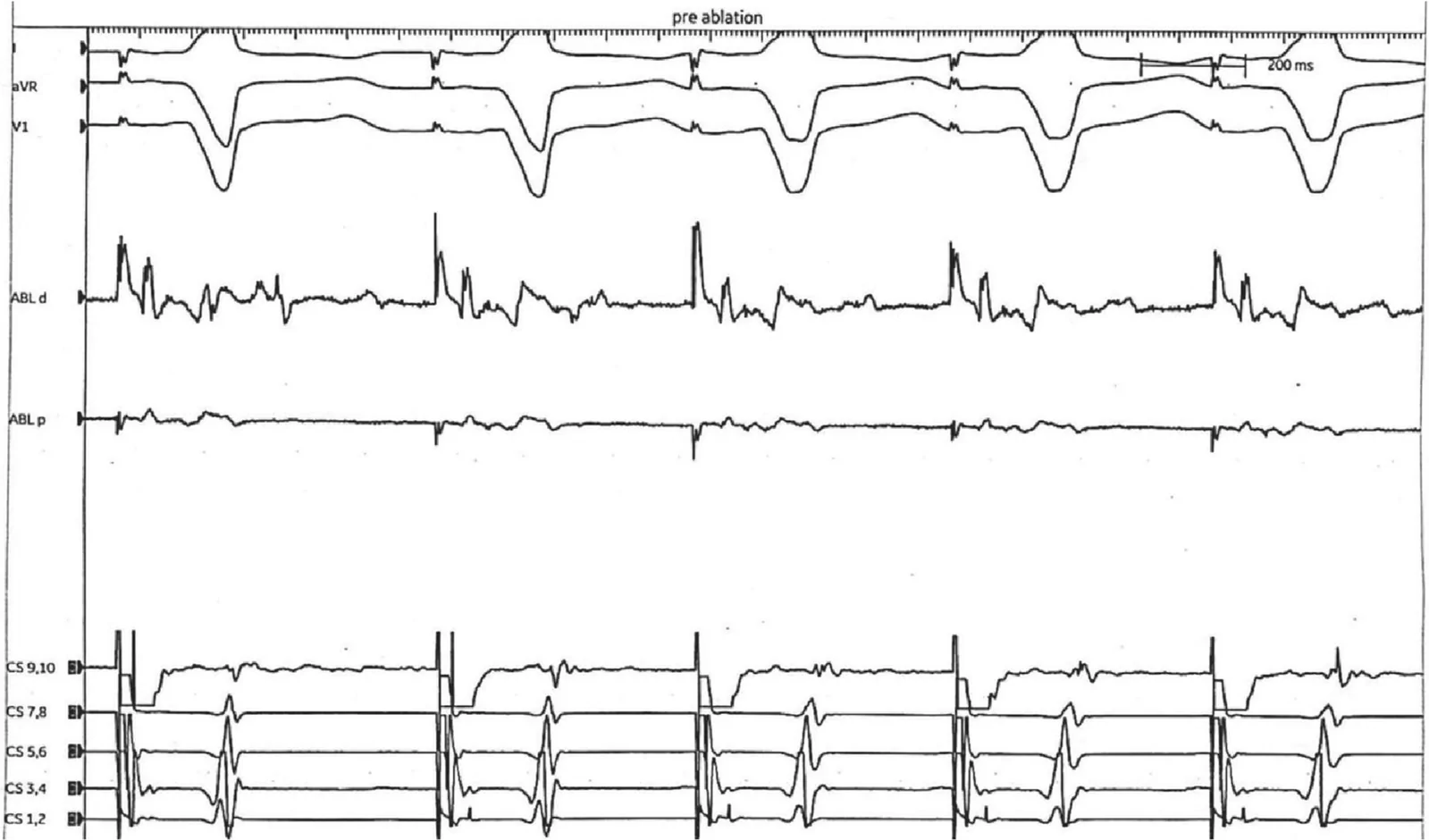
Pre-ablation intracardiac electrocardiogram from the non-coronary cusp.

**Figure 5:**
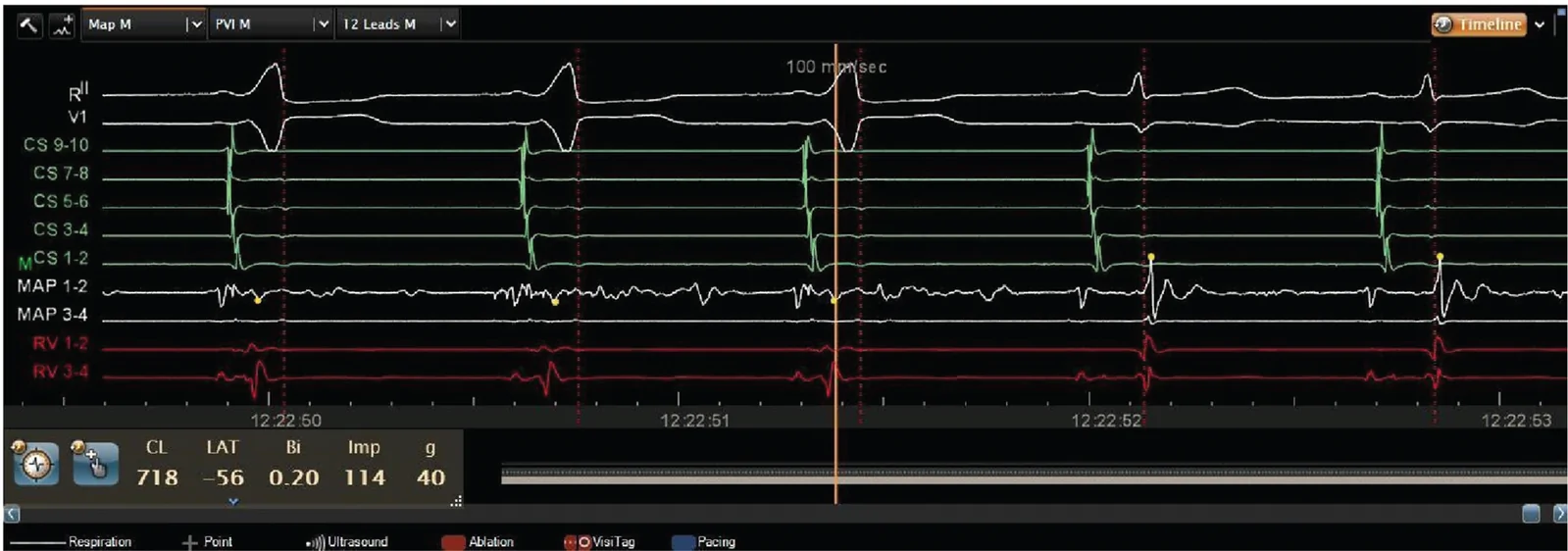
Moment of interruption of stimulus conduction through the accessory pathway. The first three beats are pre-excited, and there is an accessory pathway block on the fourth beat (without pre-excitation). The bottom of the electrogram shows the time running; radiofrequency energy was applied for 2 s until interruption of the accessory pathway.

**Figure 6:**
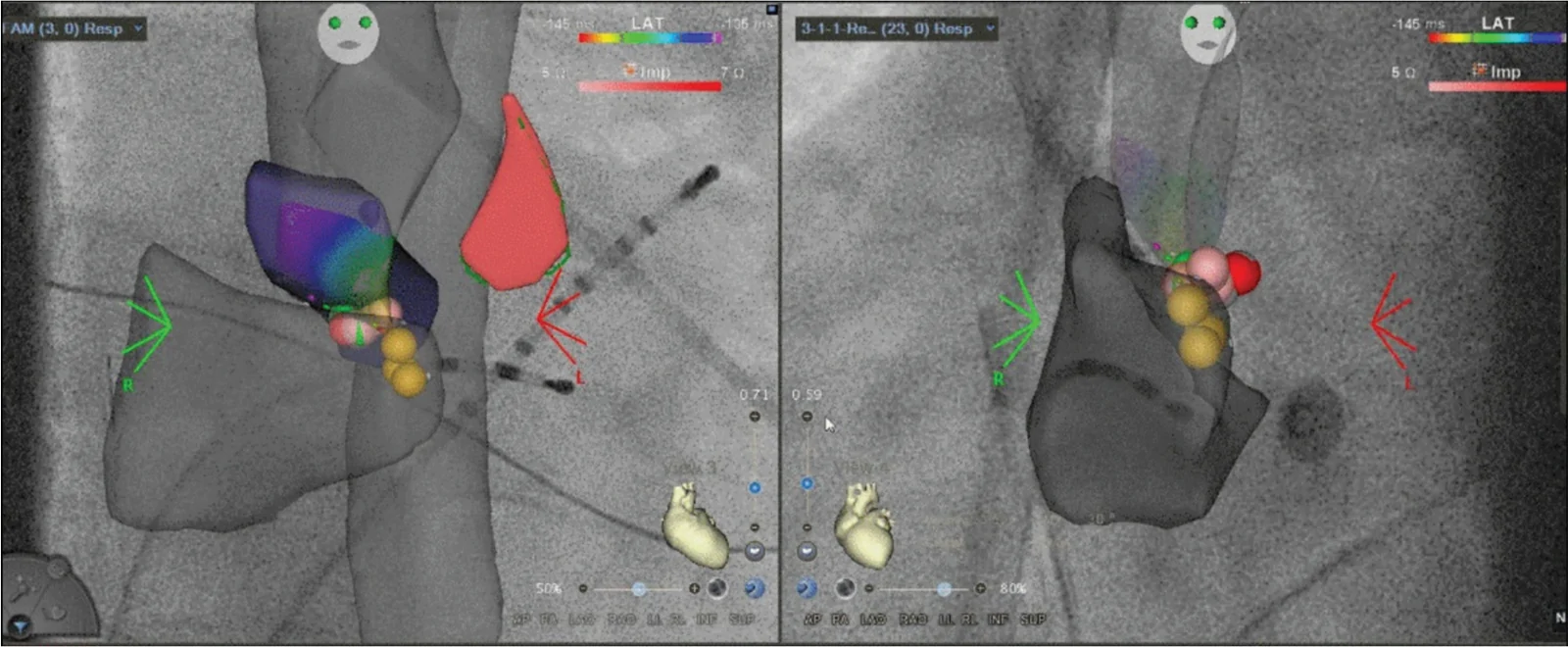
Mapping with CARTO™ and UniVu™ showing retroaortic access and the sites where radiofrequency applications were delivered in the non-coronary cusp (red points), as well as the His region (yellow points). A quadripolar catheter was positioned at the His bundle, and a decapolar catheter was positioned in the coronary sinus.

**Figure 7:**
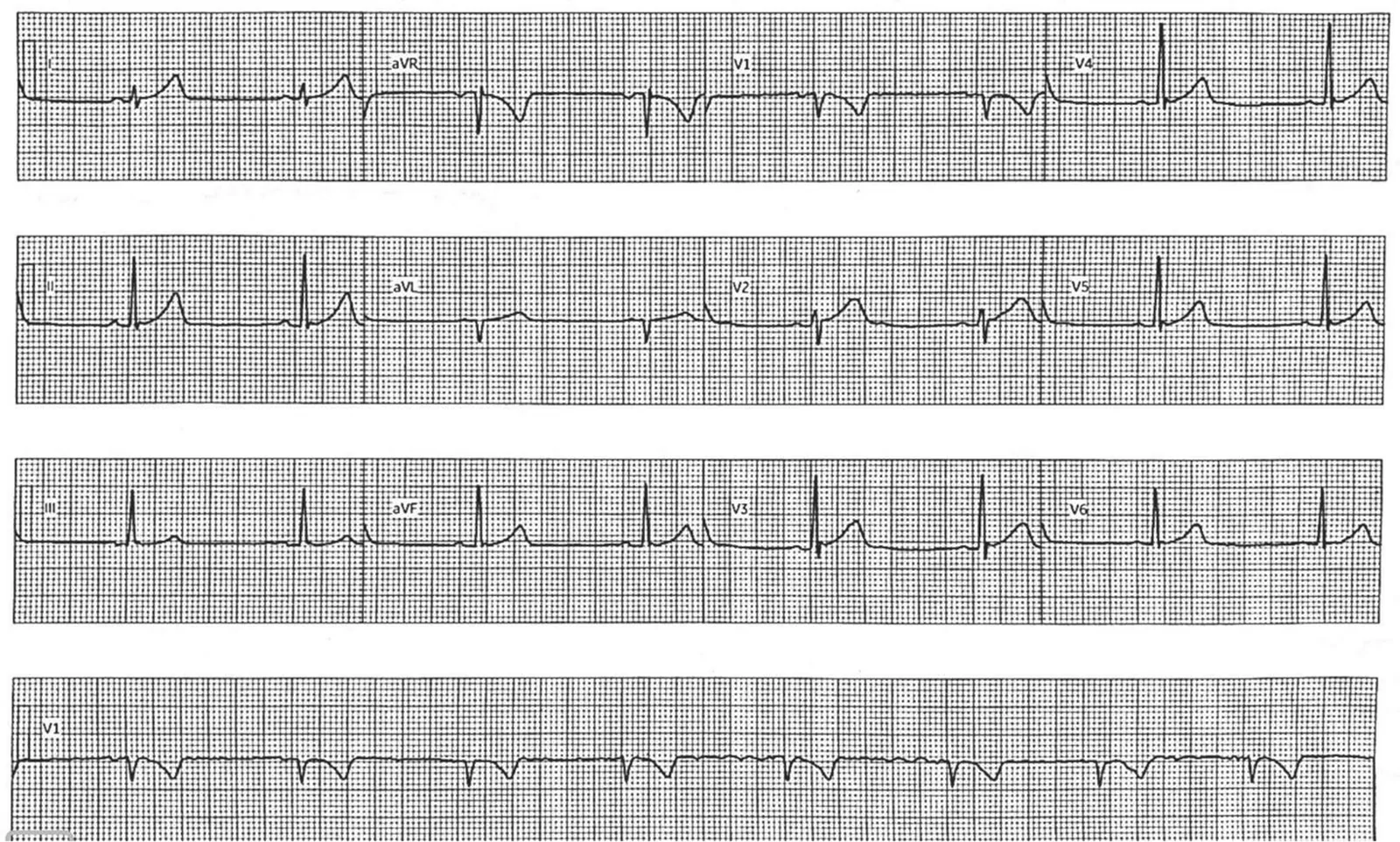
Final electrocardiogram after ablation without pre-excitation.

Given the failure of the right atrial ablation and the risk of AVB due to the close proximity to the His region, it was decided to proceed with the NCC approach. ICE was introduced and used to map the coronary arteries **(Figure 3)**. The irrigated-tip ablation catheter was positioned at the NCC via the retrograde aortic route, and mapping was done during atrial pacing from the CS at 600 ms. At the NCC, there was an AP potential with AV fusion **(Figure 4)**, distinguished by the atrial extra-stimulus protocol from the His signal. The earliest ventricular activation before the delta wave was 20 ms at the target location. Radiofrequency applications at 30 W for 20 s were applied at this site, resulting in the elimination of pre-excitation within 3 s **(Figures 5 and 6)**. Four additional lesions were delivered up to 40 W for 20–30 s per application. Twelve milligrams of adenosine was administered intravenously, which resulted in AV and VA dissociation. The post-ablation H–V interval was 48 ms. No recurrence of pre-excitation was observed during 2 h of post-procedural monitoring, and AV nodal conduction remained normal **(Figure 7)**.

## Discussion

Our case illustrates the importance of an EPS in establishing the diagnosis and guiding the optimal ablation site for PHAP in order to minimize the risk of injury to the AV node and His–Purkinje system.

Most PHAPs can be successfully ablated from the right atrium; however, in some cases, this approach may not be effective or feasible, with the presence of a high His potential amplitude (>0.1 mV) at optimal right-sided mapping, representing an excessive AVB risk. In such scenarios, ablation of PHAP via the NCC has been described as an alternative strategy.

The NCC lies immediately adjacent to both the right and left atria myocardium, superior to the central fibrous body through which the His bundle passes, and in close proximity to the ventricular myocardium inferiorly. This unique anatomical relationship allows for the presence of APs along the epicardial aspect of the NCC and supports its role as a potential ablation site, without conferring significant risk to the AV node or the His–Purkinje system.^1,2^

Major clinical studies comparing standard PHAP ablation (right anterior septal [RAS] approach) with ablation via NCC demonstrated important differences in efficacy and safety **(Table 1)**.

**Table 1:** Comparison of Clinical Trials Evaluating Para-Hisian Ablation Approaches

Study	Compared Approaches	Sample Size (Patients)	Acute Efficacy	Complications	Long-term Success
Xu et al.^3^	RAS vs. NCC	17	NCC, 92%; RAS, 42%	NCC, no complications; RAS, 33%	No recurrence (22.4 ± 15 months)
Liang et al.^4^	IVC, SVC, and NCC	55	IVC, 87%; NCC, 7%; and SVC, 6%	No complication during the perioperative period in all groups	3.6% recurrence in patients with the IVC approach. No recurrence in the NCC or SVC approach (3.2 ± 2.1 years)
Chokr et al.^5^	RAS vs. aortic cusps (including NCC)	20	Aortic cusp, 65% (7 patients as the first attempt and 6 after RAS failed)	No acute complications	No patients with successful ablation presented recurrence (27.6 ± 12.5 months)

*Abbreviations:* IVC, inferior vena cava; NCC, non-coronary cusp; RAS, right anterior septum; SVC, superior vena cava; VA, ventriculoatrial.

Xu et al.^3^ conducted a direct comparison in a cohort of 17 patients with PHAP, using either the RAS approach or the NCC approach as the initial ablation target. Ablation of NCC showed a higher acute success rate (92% vs. 42%) and a lower complication rate (0% vs. 33%) compared to the RAS approach. Long-term arrhythmia-free survival was excellent for both groups. Liang et al.^4^ evaluated 55 patients and found that most PHAPs (87%) could be ablated successfully via the inferior vena cava (IVC) approach, with only a minority requiring NCC ablation (7%). The study revealed no complications during the perioperative period in all groups and a 3.6% recurrence rate in patients who underwent the IVC approach versus no recurrence in the NCC or superior cava approach. It was concluded that NCC is not the preferred initial strategy but is valuable when right-sided ablation fails or when mapping suggests a left-sided origin. Finally, Chokr et al.^5^ provided further support that the aortic cusp (including NCC) approach was successful in 65% of cases overall and particularly effective when right-sided ablation failed. Combining both approaches yielded a high overall success rate (90%), and the choice of approach should be guided by mapping findings.

All studies used a 4-mm non-irrigated-tip ablation catheter, and the choice for the NCC approach was variable, with some cases adopting it as the first approach and others selecting it after a right-sided ablation failed. The initial power used was between 10 and 20 W for 5–10 s up to 40 W depending on the anatomic location, response of AP and AV node conduction, and the presence of junctional escapes, with a temperature limit of 55°C. Two studies performed coronary angiography in all patients to map the coronary arteries; Chokr et al.^5^ performed ablation after careful mapping at the transition area between the NCC and right coronary cusp represented by an A/V ratio of ≥1, which was previously described as a target for successful ablation by Sasaki et al.^6^ By positioning the catheter close to a reference catheter in the right atrium and parallel to the conduction system, this strategy facilitated no occurrence of AVB. It was also described by Park et al.^7^ in a series of 19 patients who underwent catheter ablation from the coronary cusps with no occurrence of AVB during NCC ablations.

In all studies, surface ECG does not appear to reliably demonstrate features that predict the success of an NCC approach; rather, the key predictors are derived from detailed electrophysiologic mapping findings, which are essential for determining the most effective ablation strategy. Liang et al.^4^ suggested that the degree of VA fusion during retrograde AP conduction at the para-Hisian region is an important mapping criterion. Pathways successfully ablated from the right atrium demonstrated well-fused local ventricular and atrial potentials in 45 of 48 cases. The lack of fusion suggests that the pathway’s ventricular insertion is deeper or more leftward, making effective right-sided ablation challenging. Chokr et al.^5^ suggested that a pre-delta time (earliest ventricular activation before delta wave onset) of <23 ms at the right septal regions predicts NCC success with high sensitivity (75%) and specificity (71.4%); conversely, a pre-delta time of >23 ms favors right-sided success.

Although the NCC approach appears to be safe with respect to AVB risk, damage of the coronary arteries has to be taken into consideration. In our case, ICE provided additional anatomical details, particularly in delineating the aortic root and the spatial relationship between the NCC and adjacent structures, including the His region. This facilitated more precise catheter positioning and may have contributed to procedural safety by helping to avoid injury to critical conduction tissue. The 2019 Heart Rhythm Society/European Heart Rhythm Association/Asia Pacific Heart Rhythm Society/Latin American Heart Rhythm Society expert consensus statement recommends ensuring a minimum distance of ≥10 mm from the coronary ostia before delivering radiofrequency energy at the aortic sinuses of Valsalva.^8^ In a series of 35 patients undergoing aortic cusp ablation, 91% were successfully treated using ICE and electroanatomic mapping without requiring coronary angiography, with the catheter tip directly visualized >1 cm from the coronary ostia in all cases.^9^ ICE offers an alternative that can obviate the need for coronary angiography in many cases. The coronary ostia can be identified by ICE and marked in the electroanatomic map for continuous real-time monitoring.

However, it is well recognized that ICE is not universally available, and its use varies significantly across centers and countries. In this context, alternative strategies for coronary artery mapping have been described, such as selective angiography performed through the ablation catheter, an innovative technique that avoids the need for additional vascular access. In this approach, contrast is injected directly through a cooled-tip radiofrequency ablation catheter at the target site, allowing assessment of the spatial relationship between the catheter tip and the coronary ostia. In a prospective study involving 12 patients, this technique enabled continuous real-time evaluation without complications or technical issues related to the ablation catheter.^10^ Another alternative is image integration, in which pre-procedural computed tomography (CT) angiography or multidetector CT images are merged with three-dimensional electroanatomic mapping, providing continuous monitoring of the spatial relationship between the catheter tip and the coronary vessels throughout the procedure.^11^

## Conclusion

Despite representing anatomical challenges, the NCC approach offers high success rates, low complication rates, and long-term freedom from arrhythmia recurrence but is generally reserved for cases where right-sided ablation is unsuccessful or mapping suggests a left-sided origin. The decision should be individualized and based on electroanatomic mapping.
